# Public Immunity: Evolutionary Spandrels for Pathway-Amplifying Protective Antibodies

**DOI:** 10.3389/fimmu.2021.708882

**Published:** 2021-12-09

**Authors:** Maya Sangesland, Daniel Lingwood

**Affiliations:** The Ragon Institute of Massachusetts General Hospital, Massachusetts Institute of Technology, and Harvard University, Cambridge, MA, United States

**Keywords:** B cells, broadly neutralizing antibodies, vaccination, immunity, evolution

## Abstract

Humoral immunity is seeded by affinity between the B cell receptor (BCR) and cognate antigen. While the BCR is a chimeric display of diverse antigen engagement solutions, we discuss its functional activity as an ‘innate-like’ immune receptor, wherein genetically hardwired antigen complementarity can serve as reproducible templates for pathway-amplifying otherwise immunologically recessive antibody responses. We propose that the capacity for germline reactivity to new antigen emerged as a set of evolutionary spandrels or coupled traits, which can now be exploited by rational vaccine design to focus humoral immunity upon conventionally immune-subdominant antibody targets. Accordingly, we suggest that evolutionary spandrels account for the necessary but unanticipated antigen reactivity of the germline antibody repertoire.

## The BCR as a Functionally Adaptive Immune Receptor

**Figure 1:**
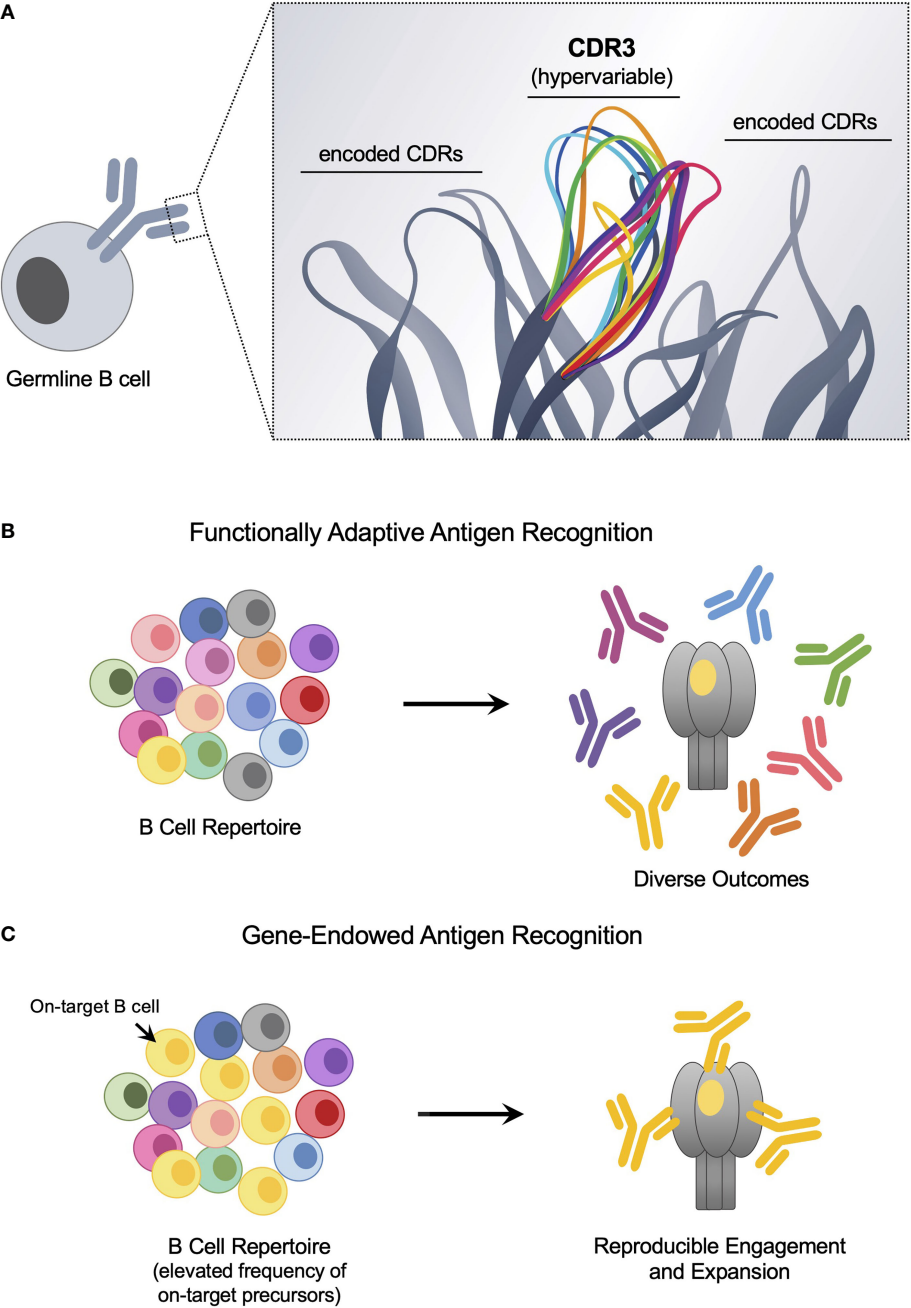
The antigen-binding surface of the germline BCR harbors functionally adaptive and ‘innate-like’ antigen targeting solutions. **(A)** The antigen-binding site of the germline BCR is formed by antibody V gene-encoded CDRs and hypervariable CDR3s that are centrally positioned. BCR diversity is a product of combinatorial assortment of the gene-encoded CDR repertoire and stochastic N-junctional diversification, which generates deep plurality in the CDR3. **(B)** As diversity is concentrated within the hypervariable CDR3, these loops are the principal source of binding energy to engage new antigen, providing functionally adaptive antigen receptor activity. However, If CDR3-centered targeting solutions are rare, then the corresponding on-target BCRs will be poorly expanded following immune challenge. **(C)** Gene-endowed antigen recognition can occur when specific encoded CDRs supply reproducible and deterministic specificity to a target epitope. This reproducibility can be exploited to ‘pathway-amplify’ otherwise immunologically subdominant vaccine responses.

It has been more than 100 years since Paul Ehrlich’s proposal of immune receptors with distinct antigen specificities (1–3), from which the B cell antigen receptor (BCR) has emerged as a cornerstone of adaptive immunity. This highly diversified ligand binding surface enables functional adaptation, facilitating the accommodation of essentially any antigen. Receptor diversity is achieved through recombination of antibody V, D and J gene segments during B cell development, resulting in a vast plurality of BCR-antigen binding sites, each unique to an individual B cell clone (4, 5). Each binding site is composed of six antigen-engaging loops or complementarity determining regions (CDRs) in which the centrally positioned hypervariable CDR3 loops on the heavy and light chains are surrounded by CDRs encoded by antibody V region genes (6–11) (**Figure 1A**). During antibody recombination, N-junctional diversification between the antibody V, D and J gene segments concentrates BCR diversity within the heavy chain CDR3 (CDRH3), which in conjunction with combinatorial assortment of the gene-encoded CDRs, results in a germline repertoire of ~10^12^ unique antigen binding sites (12–14). Hypervariable CDRH3 diversity thus enables functionally adaptive complementarity against previously ‘unseen’ molecular targets (13, 15, 16).

## Gene-Endowed BCR-Antigen Complementarity

In addition to the BCR’s adaptive immunoreceptor activity, gene-encoded CDRs have long-been hypothesized to endow hardwired affinity for specific antigen substrates (17–19). Such activity was initially observed as the constrained use of a single antibody V_H_ gene by hybridomas reactive to the head group of the eukaryotic and bacterial lipid phosphorylcholine (PC) (20, 21), a gene-encoded antigen specificity that is now recognized to impart anti-septic activity to natural antibodies, polyreactive immunoglobulins that circulate at high titer due to constitutive production (22–24). Non-random use of specific antibody V gene-encoded CDRs have since been found to underscore humoral immunity against diverse antigen input (25–34), including certain vaccine targets, which results in genetically reproducible or ‘public’ antibody responses with neutralizing activity against numerous pathogens, including influenza virus, HIV, hepatitis C virus, hepatitis B virus, yellow fever virus, respiratory syncytial virus, cytomegalovirus, SARS-CoV-2 and the malaria parasite *Plasmodium falciparum* (27, 35–56). Such responses are considered deterministic and ‘public’ because the structure of the antibody binding site is constrained to specific gene-encoded CDRs, enabling reproducible reconstruction of the same antibody within different individuals. While gene-encoded CDRs are structurally limited to a few ‘canonical’ loop conformations (11, 57, 58), they are not functionally redundant, as we have recently demonstrated using HC2 humanized mice, where antibody responses are constrained to single human V_H_ genes but also bear unconstrained human-like CDRH3 diversity (59–61). These studies demonstrate that specific antibody V_H_ gene input to the repertoire is required for germline B cells to accommodate and respond to select epitopes and/or entire antigen geometries *in vivo* (59–62). Hence, BCR substrate specificity can be both functionally adaptive (CDRH3 dominant) and genetically programmed (antibody V gene constrained). The latter forms the basis of gene-endowed B cell antigen recognition.

B cell receptor signaling is also tightly integrated with innate immune signals. After ligation of Toll-like receptors, conventional innate immune receptors for pathogen-associated molecular patterns (PAMPs), B cell proliferation also requires BCR-dependent signaling to occur in parallel (63), consistent with BCR triggering as a tonic survival signal (64, 65).

## Gene-Endowed Antigen Recognition as Reproducible Substrate for Germline Stimulating Vaccines

Hypervariable viruses such as HIV and influenza continue to defy conventional vaccine approaches (66–69). Indeed, a major obstacle to eliciting broadly protective responses is largely attributed to the fact that the surface antigens of these viruses establish complex immunodominance hierarchies that effectively distract the host antibody response away from functionally conserved sites of vulnerability, both following infection or vaccination (62, 70–75). It is well established that the strength of an antibody response against a given epitope is, in part, a function of the proportion and cognate affinities of the corresponding on-target BCRs present within the germline B cell repertoire (59, 60, 62, 76–82). On-target BCRs refer to germline antibodies bearing affinity for a desired epitope (e.g. a conserved site of vulnerability on the surface glycoproteins of HIV or influenza virus), whereas off-target BCRs engage different epitopes on the same antigen (the otherwise hypervariable features in the case of ‘vaccine resisting’ viruses). Immunodistraction by HIV and influenza viral antigens can occur when the on-target BCRs fail to compete with off-target BCRs, allowing the latter to dominate affinity maturation within B cell germinal center reactions, which subsequently carries over into downstream B cell memory and serum antibody responses (59, 60, 62, 76–83). Germline stimulating vaccines, particularly in the HIV space, have focused on engineering enhanced immunogen affinity to specific on-target BCRs that are known precursors for broadly neutralizing antibodies (bnAbs) (76, 77, 79, 84–100). These approaches have provided proof of concept for selective priming and expansion of human B cell lineages, both within human BCR-knock in mice (84–88, 91–93, 95, 97, 99–101) and within mice bearing adoptively transferred bnAb precursors at diluted B cell frequencies (76, 77, 79, 80, 89, 98). Consequently, a leading germline stimulating HIV vaccine candidate is currently under clinical evaluation (Clinical Trials Identifier: NCT03547245).

Importantly, gene-endowed BCR antigen recognition provides natural reproducibility in epitope-targeting that can bolster the proportion of on-target BCRs present within the antigen naïve B cell repertoire (**Figures 1B, C**). The centrally positioned CDRH3 is by definition hypervariable (12–15). Thus bnAb precursors with CDRH3-dominant contacts will be difficult to selectively target and expand by germline stimulating immunogens, particularly if they are rare; although recent advances in protein engineering have made exciting headway in addressing this issue (80, 102). By contrast, the paratopes of human antibody gene-endowed bnAbs, both against HIV and influenza virus, are dominated by genetically encoded CDRs (35, 37, 38, 45, 53, 103, 104), indicative of a reproducible, deterministic source of on-target-specificity that may be expanded by vaccination. Most germline stimulating concepts have therefore focused on bnAb pathways that involve antibody gene-endowed antigen contact, as reflected by the heavy emphasis on vaccine-elicitation of human VRC01-class bnAbs which rely on usage of the antibody V_H_ gene IGHV1-2*02 (76, 77, 79, 84–101).

Using the HC2 mouse system, we have shown that individual human V_H_ genes do indeed selectively endow the polyclonal BCR repertoire with natural germline specificity for select epitopes (59, 60, 62). We found that in contrast to other human V_H_ genes, IGHV1-69*01 endows the otherwise unconstrained human-like BCR repertoire with the capacity to engage a conserved site of vulnerability on the surface of the major influenza viral spike protein hemagglutinin (59, 60, 62). This gene-endowed affinity provided substrate for reproducible pathway-amplification of broadly protective influenza bnAb responses which were elicited using a single rationally designed hemagglutinin immunogen (59, 60, 62). Importantly, the capacity to pathway-amplify influenza bnAbs was directly proportional to the frequency of IGHV1-69*01 B cells present within the repertoire, a parameter that could be genetically diluted in the HC2 system (59). This dose response-relationship further reveals a deterministic/gene-programmed antigen recognition activity by B cells and demonstrates how public humoral immunity can serve as a reproducible template for selectively amplifying antibody responses that engage otherwise unseen vaccine targets.

## Gene-Endowed Antigen Recognition as Spandrels of Evolution

How might we place vaccine-amplifiable public immunity within the context of evolution? Undoubtedly the repertoire was shaped by positive selection (17–19, 27), and it is tempting to speculate that specific antibody V genes were retained to enable recognition against specific pathogens or groups of pathogens. However, it will be difficult to demonstrate that any one particular antibody V gene sequence was selected due to an immediate fitness benefit, as would be required for an evolutionary adaptation (105–107). Moreover, unless public bnAb responses (such as those against HIV and influenza virus) are selectively triggered and pathway-amplified by rationally designed vaccines, their benefit to combating infection at ‘steady-state’ is not clear. Gene-endowed antigen recognition also enables public human neutralizing antibody responses against more recently emerged pathogens, such as HIV (35, 45, 108) and SARS-CoV-2 (50, 54, 55), arguing against an evolutionarily adaptive relationship. Indeed, using HC2 humanized mice, we demonstrated that the IGHV1-2*02 V_H_ sequence utilized in public VRC01-class HIV bnAbs, also endows the germline BCR repertoire with the capacity to engage the conserved saccrolipid core of LPS, a ‘primordial’ but key surface antigen from gram-negative bacteria (61). This underscores the notion of broad germline utility (27, 28, 32), which we propose is best placed within the framework of evolutionary spandrels.

**Figure 2:**
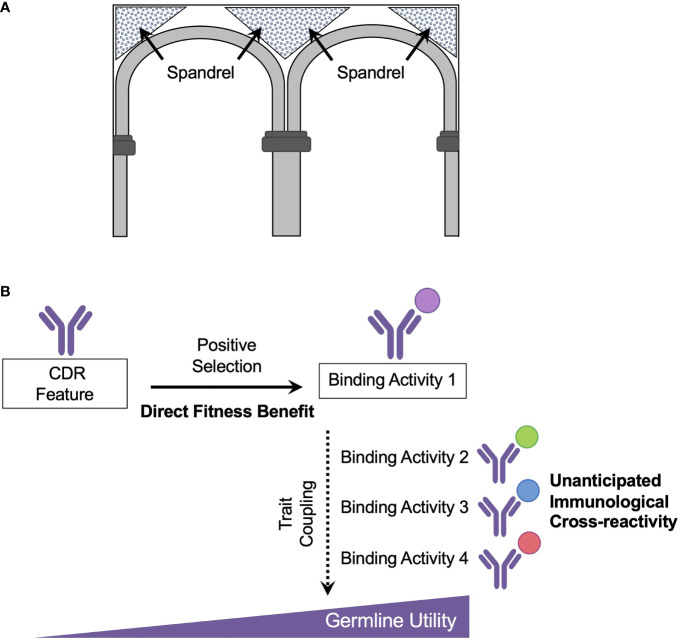
Recognition of new antigen as an evolutionary spandrel **(A)** A spandrel is an architectural by-product formed by adjacent rounded arches within a rectangular frame. **(B)** Germline antibody reactivity and utility is proposed to emerge as a set of spandrels or secondary ‘unintended’ consequences of positive selection on the germline antibody repertoire. In this scheme, the antibody repertoire was initially shaped by positive selection for combating disease-causing pathogens. A key evolutionary adaptation in this process was the development of recombinatorial lymphocyte immune receptors, which appeared in vertebrates ~500 mya ago. However, germline antibody reactivity to new, different, antigen cannot be considered as an evolutionary adaptation unless they result in an increase in inclusive fitness. Rather the capacity for reactivity to novel antigen (hen egg lysosome, ovalbumin, vaccine antigens) is more appropriately framed as spandrels or unanticipated biproducts of the primary adaptations which shaped the antibody repertoire over evolutionary time. A case example are genetically encoded CDRs which show reproducible usage for engaging bnAb targets on ‘historical’ viruses such as influenza virus and on more recently emerged viruses such as HIV and SARS-CoV-2.

A term famously borrowed by Gould and Lewontin, a spandrel is an unplanned architectural feature that forms as an engineering constraint during the construction of domed cathedrals and was used to designate evolutionary characteristics that arise in the absence of direct selection and are byproducts of another ‘decision’ in design (109, 110) (**Figure 2A**). The importance of evolutionary spandrels in shaping biological form and function has been observed across many scales (107, 110–114). At the animal level, a classical example is in female spotted hyenas, where muscularized genitalia has been viewed as an evolutionary byproduct resulting from elevated testosterone, an endocrine adaptation associated with superior size and female dominance (110). At the molecular scale, evolutionary spandrels have accounted for structural coupling between protein folding and emergence of affinity to novel binding partners (114). Such coupled traits can appear in the absence of an immediate fitness benefit and may provide ‘unanticipated’ utilities. We suggest that germline antibody responsiveness, and hence the potential for ‘unplanned’ immune reactivity to previously unseen antigen, is explained by this same evolutionary principle (**Figure 2B**).

Recombinatorial lymphocyte immune receptors appeared in vertebrates ~500 mya ago and likely emerged as a key evolutionary adaptation (or series of adaptations) leading to positive selection (115–117). In jawed vertebrates, VDJ recombination supplies germline BCR diversification, and in jawless vertebrates, the functionally analogous variable lymphocyte receptors (VLRs) are diversified *via* recombination of leucine-rich-repeat gene modules (118, 119). However, germline reactivity and cross-reactivity to new ‘unanticipated’ antigens (e.g. present-day vaccines) will be an unplanned consequence of these diversification processes, which have been pre-shaped over evolutionary time. Thus within an evolutionary framework, the capacity for germline antibody responsiveness and cross-reactivity to incoming antigen should be considered as a side effect or spandrel. Included here will be the public antibody contact solutions that endow for genetically reproducible human bnAb responses that require rational vaccine engineering for selective triggering and pathway-amplification (**Figure 2B**). Unless the specific germline encoded response to antigen can be demonstrated as evolutionarily adaptive, i.e. provided an increase in inclusive fitness (105–107), its emergence is best described as an unanticipated utility.

Evolutionary spandrels as a means of developing ‘unplanned’ utility in antigen recognition may also have fueled the “tonic” BCR-dependent signaling required to coordinate B cell survival and proliferation with activation signals triggered by PAMPs and T cell ligands (63–65). Such coupling between innate and adaptive immune reactions will be critical for both adjuvating B cell activation and linking T cell help during the humoral response.

## Perspective

Due to its genetic reproducibility, gene-endowed antigen recognition can increase the likelihood of engaging certain epitopes or antigenic configurations, providing a framework to rationally overcome the immunological subdominance of some antigenic targets. Proof-of-concept studies in the pre-clinical space indicate that this unconventional mode of B cell antigen engagement can be exploited to pathway-amplify genetically reproducible antibody targeting solutions against otherwise difficult to perceive sites of vulnerability on vaccine-resistant pathogens and is thus poised for more comprehensive clinical evaluation. The capacity for pathway-amplification most likely arose as an evolutionary spandrel or secondary consequence of how the antibody repertoire was shaped over evolutionary time. The evolutionarily non-adaptive manner through which spandrels emerge will likely ensure continued discovery of gene-endowed antibody responses against specific vaccine targets, and as a consequence, the corresponding publicly reproducible antibody development pathways that may be judiciously activated and amplified by vaccination.

## Author Contributions

MS and DL wrote the manuscript. All authors contributed to the article and approved the submitted version.

## Funding

DL was supported by the NIH (R01AI137057, DP2DA042422, R01AI124378, R01AI153098, R01AI155447), the Harvard University Milton Award, and The Gilead Research Scholars Program Institute. MS was supported by an NIH fellowship (F31Al138368).

## Conflict of Interest

The authors declare that the research was conducted in the absence of any commercial or financial relationships that could be construed as a potential conflict of interest.

The handling editor declared a past collaboration with one of the authors DL.

## Publisher’s Note

All claims expressed in this article are solely those of the authors and do not necessarily represent those of their affiliated organizations, or those of the publisher, the editors and the reviewers. Any product that may be evaluated in this article, or claim that may be made by its manufacturer, is not guaranteed or endorsed by the publisher.
